# Epigenetic DNA Methylation Linked to Social Dominance

**DOI:** 10.1371/journal.pone.0144750

**Published:** 2015-12-30

**Authors:** Kapa Lenkov, Mi H. Lee, Olga D. Lenkov, Andrew Swafford, Russell D. Fernald

**Affiliations:** Biology Department, 371 Serra Mall, Stanford University, Stanford, CA 94305–5020, United States of America; University of Basel, SWITZERLAND

## Abstract

Social status hierarchies are ubiquitous in vertebrate social systems, including humans. It is well known that social rank can influence quality of life dramatically among members of social groups. For example, high-ranking individuals have greater access to resources, including food and mating prerogatives that, in turn, have a positive impact on their reproductive success and health. In contrast low ranking individuals typically have limited reproductive success and may experience lasting social and physiological costs. Ultimately, social rank and behavior are regulated by changes in gene expression. However, little is known about mechanisms that transduce social cues into transcriptional changes. Since social behavior is a dynamic process, we hypothesized that a molecular mechanism such as DNA methylation might play a role these changes. To test this hypothesis, we used an African cichlid fish, *Astatotilapia burtoni*, in which social rank dictates reproductive access. We show that manipulating global DNA methylation state strongly biases the outcomes of social encounters. Injecting DNA methylating and de-methylating agents in low status animals competing for status, we found that animals with chemically increased methylation states were statistically highly likely to ascend in rank. In contrast, those with inhibited methylation processes and thus lower methylation levels were statistically highly unlikely to ascend in rank. This suggests that among its many roles, DNA methylation may be linked to social status and more generally to social behavior.

## Introduction

Social status hierarchies are ubiquitous in vertebrate social systems [1], including humans [2], and social rank influences quality of life dramatically in social groups. High-ranking individuals have greater access to resources, including mating prerogatives that have a positive impact on their survival, reproductive success and ultimately their health. In contrast low ranking individuals typically have limited reproductive success and may experience lasting social and physiological costs of low status. Given an opportunity to ascend in social status during changing physical and/or social conditions, individuals must respond rapidly to exploit such chances. In many species, biological responses to social challenges such as conflicts over dominance result in modifications of behavioral and physiological phenotypes. Here we asked whether epigenetic mechanisms might play a role in modifying status related behaviors and phenotypes. To do this, we used an African cichlid fish, *Astatotilapia burtoni*, in which male social rank is determined by successful aggressive behavior and dictates reproductive access [3]. To test the role of epigenetic changes, we manipulated the DNA methylation states and showed such manipulations significantly biased the outcomes of aggressive social encounters among males.

Although evolution shapes phenotypes via genomic modifications over long time scales, epigenetic processes can change gene expression patterns rapidly without altering underlying DNA sequences. Epigenetic changes include histone acetylation, histone methylation, and DNA methylation [4]. DNA methylation, which is the covalent addition of a methyl group to a cytosine base, can occur in minutes to hours in adult animals and these changes are reversible [5]. In addition, DNA methylation can have significant effects on learning and memory in vertebrates [6], caste in insects [7], and the behavioral shift from hive work to foraging in honey bees [8]. These data suggest that epigenetic changes may have multiple roles in altering social behavior. Specifically, in rats, DNA methylation induced behaviorally through maternal care can mediate the onset of stress tolerance [9]. The mechanism identified was that maternal licking and grooming changed the levels of methylation at the glucocorticoid receptor gene promoter in rat pup hippocampus. Cross-fostering pups to a dam with low levels of maternal care reversed the induced differences in DNA methylation state of the glucocorticoid receptor gene promoter.

To identify the potential role of DNA methylation in regulating social status, we used a cichlid fish model system where rank is tightly regulated by social interactions [10]. *Astatotilapia burtoni* males live in Lake Tanganyika, east Africa, as either of two distinct, *reversible* phenotypes: 1) Dominant (D) males that have bright coloration, are reproductively capable, and defend spawning territories and; 2) non-dominant (ND) males that have a dull grey coloration blending with the substrate, school with females, and are not reproductively active. In response to specific permissive social and environmental situations, animals can and do switch between these behavioral phenotypes in minutes and change physiological characteristics essential for their new status over minutes to days [11]. In this species, as in many that defend resources, males establish social rank by engaging in highly aggressive behaviors towards other males and reproductive behaviors towards females [12].

The changes in *A*. *burtoni*, social status causes changes in the reproductive control system [13]. As in all vertebrates, the brain-pituitary-gondal axis regulates reproduction originating in the hypothalamus where gonadotropin releasing hormone (GnRH1) containing neurons deliver the decapeptide to the pituitary to control gonadal state. These neurons increase in size (8X) within a few days and several other well-described changes in the reproductive axis occur as well [14]. The GnRH1 neurons in reproductively competent D males are connected via gap junctions to produce the necessary pulsatile output [15]. Although we do not yet fully understand the mechanisms underlying all these events, based on previous work, we predicted that the GnRH1 neurons would increase their production of GnRH1 mRNA as ascent to social dominance begins.

## Materials and Methods

### Experimental Design

The overall goal of these experiments was to test whether modifying the methylation state of animals could influence the outcome of fights over social dominance in the male African cichlid fish, *A*. *burtoni*. Animals were reared as socially suppressed by larger conspecifics from birth so they had never been dominant (Fig A in S1 File). When two such males from separate rearing tanks, matched for size and color were placed in an aquarium that could sustain only one territory (Fig B in S1 File), fighting began almost immediately. In a relatively short time (<< 30 minutes), one male became dominant based on his behavior over the other male. In all cases, dominance was established quickly, consistent with observations in the natural habitat (Fig B in S1 File). This phenotypic change is evident from the dramatic changes in body coloration and in aggressive behaviors towards the fleeing non-dominant male who had lost the encounter. The non-dominant male typically flees or remains in the upper part of the water column. The winning male then directed reproductive behaviors towards females while continuing to aggressively threaten the non-dominant male, consistent with observations in the natural environment [10].

#### Behavioral analysis

Several broods of juvenile *Astatotilapia burtoni* males, were reared in tanks containing 4 large dominant males for a minimum of 2 months during which time these animals could not become dominant due to aggression of the dominant males [16] (Fig A in S1 File). All animal experiments were approved by Stanford University’s Institutional Animal Care and Use Committee (IACUC protocol 9882). Pairs were matched for size to within 0.2 cm in length and within 0.5g. Size-matched pairs (5.9 ± 0.2 cm; 5.0 ± 0.5g) of such never-been dominant (NBD) males were selected from separate rearing tanks and IP injections were performed with either the vehicle or one of two drugs: L-methionine or zebularine (200μg/g body weight). Individuals that injected the substances as well as those who ultimately scored the behavior were blind to treatment group. Immediately after injection, males were placed together in a 120 L tank with three small females for five days. Smaller male and female fish from community aquaria were placed across a clear barrier on both sides of the test males, to provide an incentive for competition between the males to become dominant and to allow courting behaviors to occur in the general repertoire of aggressive behaviors. (Fig B in S1 File).

Behaviors of subject animals were video recorded daily and assessed using a previously established scoring paradigm to quantify characteristically dominant behavior and characteristically non-dominant behavior in males [17]. The following behaviors are considered dominant behaviors: Approach, Chase, Frontal Threat, Lateral Display, Border Fight, Court/Quiver, Lead/Waggle, Dig, Spawn site entry, Bite, Chafe. Fleeing was counted as a non-territorial behavior. Behavioral data was analyzed using ANOVA. After animals were sacrificed by rapid cervical transection, the relative gonad size was measured (gonadosomatic index; GSI = [gonad mass/body mass] x 100). ND animals had a GSI between 0.2 and 0.6, while D animals have a GSI between 0.4 and 0.8.

Microdissected brain tissue was processed to measure methylation via bisulfite modification (EZ DNA methylation direct kit, Zymo Research, Irvine, CA) according to manufacturer’s instructions. Primers were directed against CpG-rich regions in the promoter and coding region of the *A*. *burtoni GnRH1* gene (Genebank accession number AF076961). Primers were designed so they did not contain any CpG dinucleotides, which meant that methylated and unmethylated sequences were amplified with the same efficiency (EpigenDX, Hopkinton, MA). The sequencing template was prepared using the standard protocol (Vacuum Prep Tool, Biotage, Uppsala, Sweden) as adapted for use in the Stanford Genome Technology Center [18]. Briefly, for each PCR amplification, one inner primer was biotinylated at the 5’ end at allow for immobilization of the amplicon. 15–20 μl of each biotinylated PCR product were combined with and immobilized on streptavidin coated sepharose beads (Dynabeads M280, Dynal, Oslo, Norway). The immobilized DNA was treated with sequential washes of 70% ethanol, 0.2 M NaOH (for denaturation of the DNA), and TA buffer. The single-stranded DNA target was then hybridized to specific sequencing primers. Pyrosequencing was performed using the PyroMark Q96 DNA sequencing system (Qiagen, Hilden, Germany) according to manufacturer’s instructions.

## Results

**Fig 1:**
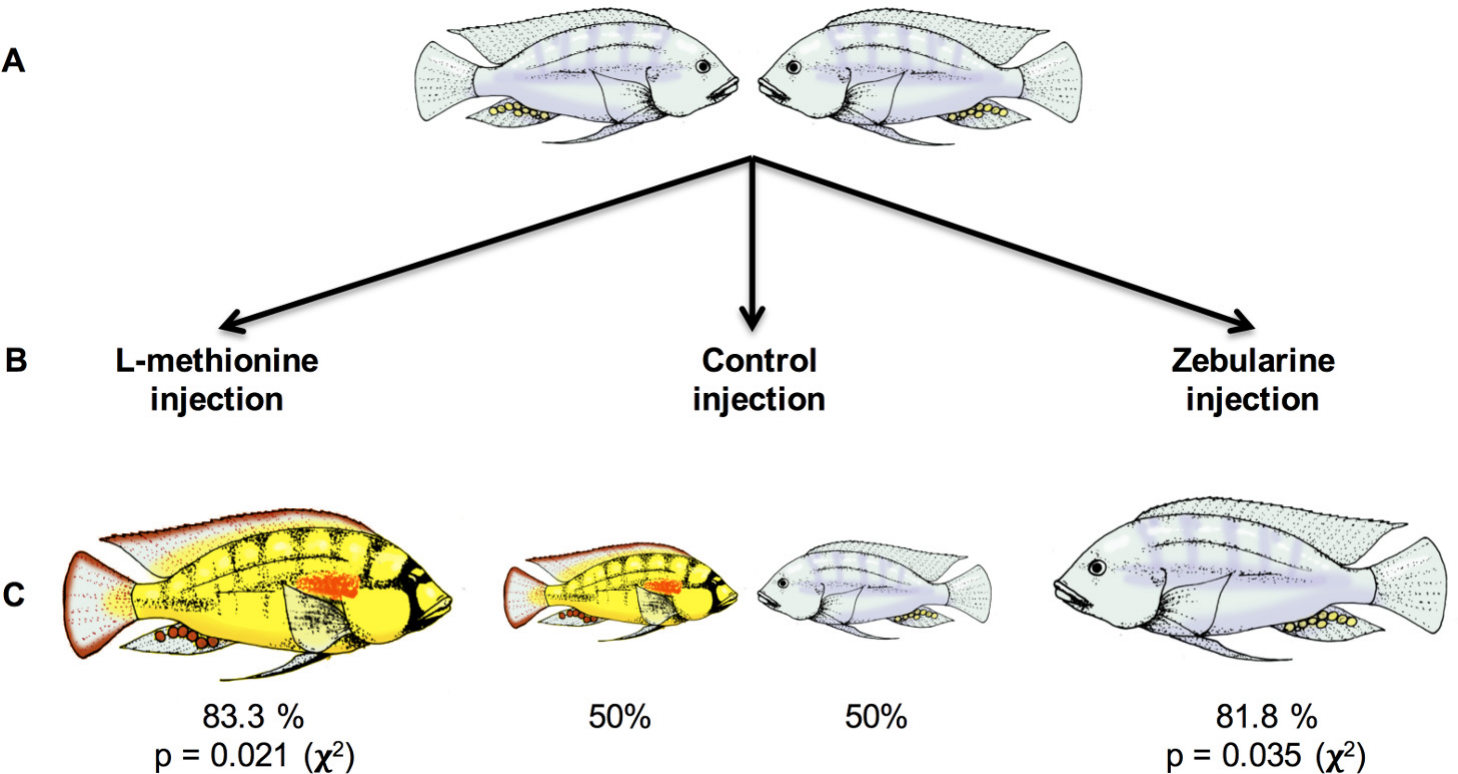
(A) Animals that had never been socially dominant were divided into three groups of size matched pairs. (B) In control animals, both members of the pair received vehicle injections (N = 10 pairs), in experimental animals, in one group (left) one member of the group received L-methionine (N = 12) and the other received vehicle control (N = 12), while in the other group (right), one member of the pair received zebularine, (N = 11) and the other received vehicle control (N = 11). (C) Animals injected with L-methionine were significantly more likely to become socially dominant, while those injected with zebularine were significantly more likely to remain non-dominant.

We measured the effect of DNA methylation modifying agents on the outcome of fights that distinguish socially dominant from non-dominant males. To do this, we compared the relative success of non-dominant animals becoming dominant between two treatments (Fig 1).

We pharmacologically manipulated the DNA methylation state and measured its effect on the outcome of such dominance fights. To induce methylation, we used L-methionine, which acts as a methyl donor and to inhibit methylation, we used zebularine [*1-(beta-D-ribofuranosyl)-1*,*2-dihydropyrimidin-2-one*], a cytidine analog that inhibits DNA methyltransferases (DNMTs) and has been shown to reverse the effects of methylation, reactivating a previously silenced gene [19]. Control animals received vehicle injections (30% DMSO in distilled water; see Supporting Information).

**Fig 2:**
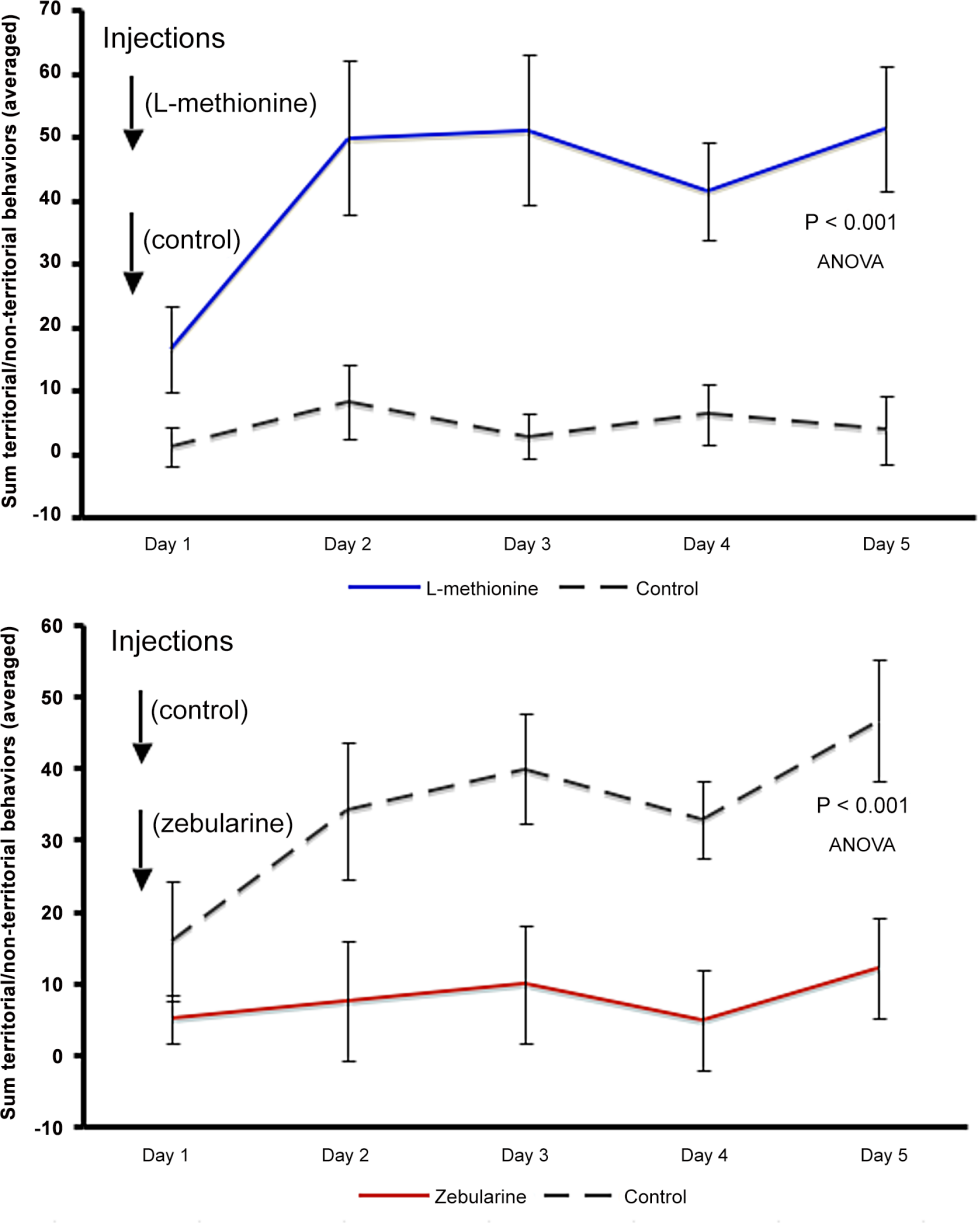
Average number of territorial acts with non-territorial behavioral acts subtracted (per 5 min) of dominant (upper line in each graph) and non-dominant animals (lower line in each graph). (A) Paired L-methionine (blue line) and vehicle-injected (gray dashed line) animals (2N = 24). There is a significant difference between methionine-injected and control-injected animals F(1,110) = 59.28, p = 6.37 x 10^−12^. There was no significant difference in behavior between individual observation days (F(4,110) = 2.32, p = 0.062) or significant interaction between injection treatment and individual days (F(4,110) = 1.52, p = 0.20). (B) Paired zebularine (red line) and vehicle injected (gray dashed line) animals (2N = 22). There was a significant difference between zebularine-injected and control-injected animals F(1,100) = 29.60, p = 3.79 x 10^−7^. There was no significant difference in behavior between individual days (F(4,100) = 1.76, p = 0.14), or significant interaction between injection treatment and individual days (F(4,100) = 0.70, p = 0.59).

Animals injected with L-methionine were much more likely to become socially dominant (p = 0.021, X^2^ test; N = 12 pairs) while animals injected with zebularine were much more likely to remain non-dominant (Fig 1; p = 0.035, X^2^ test; N = 11 pairs). Behavior of all animals was filmed and scored by observers’ blind to the experimental conditions. As seen in Fig 2, there is a striking difference between fish in response to the injections of a methyl donor (L-methionine) and a methylation inhibitor (zebularine). Animals injected with L-methionine became D within the first day, exhibiting the coloration and behavioral phenotype rapidly. In contrast, the animals injected with zebularine did not become D but remained ND, their coloration remained drab and their behavior was restricted to fleeing and hiding. In control conditions, there were no qualitative or quantitative differences in the behavior in fish between those that became dominant with or without methionine injections (Fig C in S1 File methionine; Fig D in S1 File zebularine). Similarly, no differences were observed in the behavior of fish that remained non-dominant with or without zebularine injections (Figs B and C in S1 File; p > 0.05, ANOVA). That is, none of the substances injected on their own significantly altered the behavioral activities of the animals but the outcome depended on the social interaction.

Since either of these two chemicals potentially could have had global effects on the genome, we asked whether the effects of injecting methionine and zebularine were limited to a subset of genes or if they altered DNA methylation more widely. We assessed methylation state following treatments by immunolabeling methylated nuclei. We examined brain sections from hypothalamic regions since these brain nuclei are known to be implicated in social behaviors including reproduction [20]. Comparing brain sections of L-methionine and vehicle treated D animals and zebularine and vehicle-treated ND animals, we found no significant difference in staining intensity between these treatment groups, suggesting that global genomic methylation levels were unaffected by social status or L-methionine or zebularine treatment (Figs E and F in S1 File).

To assess specific molecular consequences of the methylation treatments, we measured the methylation profiles with bisulfite sequencing of the gonadotropin releasing hormone (*GnRH*) gene five days after the animals achieved a stable social status. We have previously shown that GnRH mRNA expression responds dramatically and rapidly to changes as a function of change in social status [21] As noted above, when an animal ascends from ND–> D, one important consequence is that in ~2 days, the male becomes reproductively competent, a process controlled by the brain via the hypothalamic-pituitary-gonadal (HPG) axis in this cichlid as in all vertebrates. As noted above, the apex of the HPG axis, is *GnRH* that produces GnRH1, a decapeptide, which travels to the pituitary where it regulates gonadal function. In a previous study, we showed that within 20 minutes after a male ascends from ND -> D, *egr-1*, an immediate early gene and putative GnRH1 transcription factor [22] is upregulated [23]. Within an hour, this change was accompanied with dramatically increased GnRH1 mRNA production [23].

**Fig 3:**
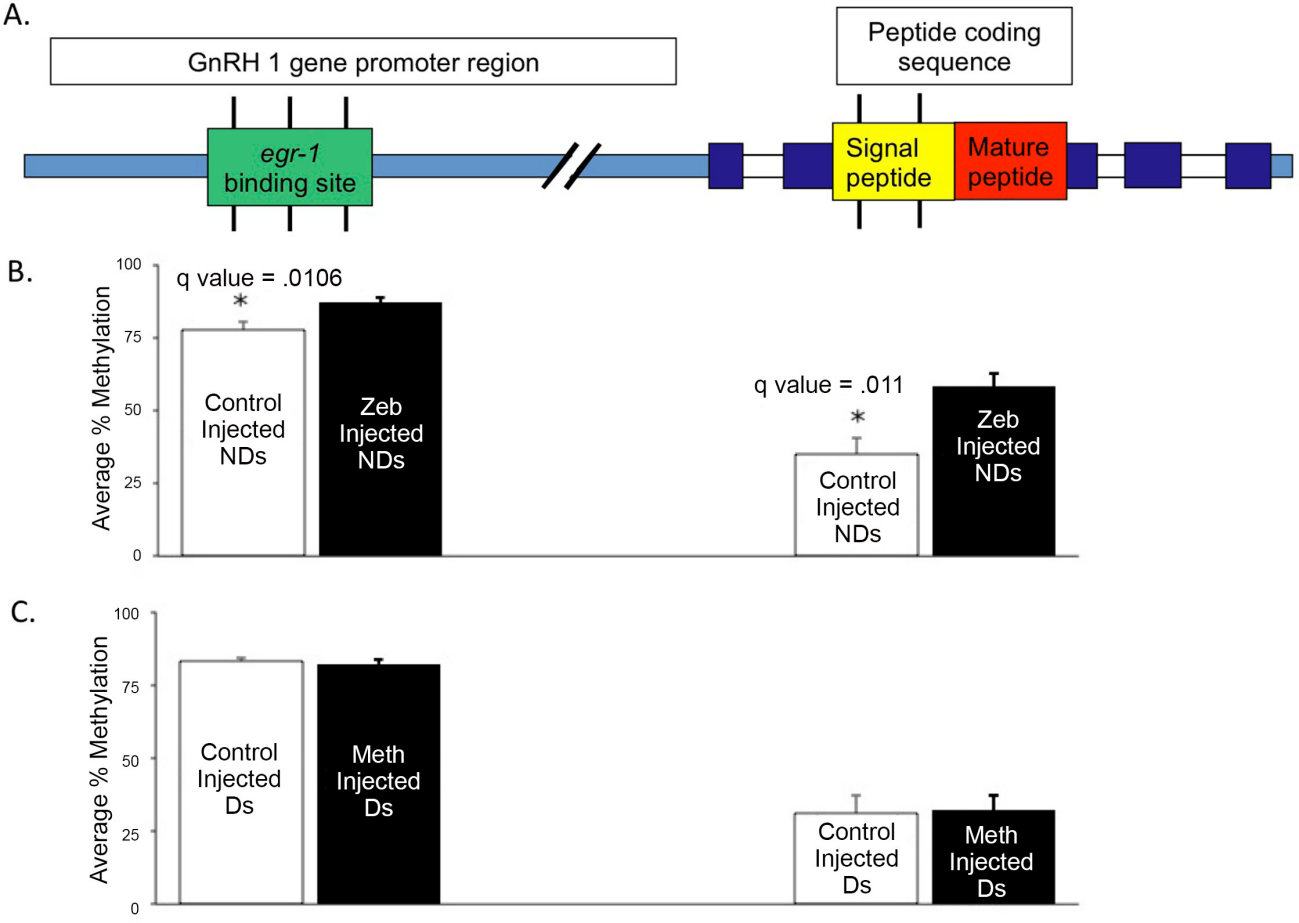
Differential methylation at CpG sites within functional genomic regions of *GnRH1* in the pre-optic area (POA) in zebularine-injected, L-methionine-injected and control-injected dominant (D) and non-dominant (ND) males 5 days after initial injection. **A**. Methylation was assayed in two locations on the *GnRH1* gene; in the promoter region within the binding site of *egr1* (3 CpG sites indicated by single vertical bars within the green rectangle) and in the coding region in the sequence of the signal peptide (2 CpG sites indicated by single bars within the yellow rectangle). **B**. Average methylation levels in *GnRH1* genomic regions of zebularine-injected ND males that show significantly higher average methylation levels than control-injected ND males in the *egr-1* binding site located in promoter region of *GnRH1*. (*q-value < 0.05; n = 3–4 per group). **C**. Average methylation levels in *GnRH1* genomic regions of methionine-injected ND males are not significantly different from those of control injected animals (q-value > 0.05 for all groups; n = 3–8 per group). Statistical significance for each of these regions was calculated by determining combined Fisher’s p values and then calculating q values estimating the multiple-testing-corrected false discovery rate (see Methods in S1 File for complete description). Error bars represent s.e.m.

At the sacrifice of the animals 5 days after the status change, we identified differential methylation within the putative *Egr-1* binding site in the GnRH promoter (Fig 3A). There were significant increases in methylation within this CpG site in response to zebularine injection (Fig 3B) as well as an increase in methylation at one position in the GnRH1 coding region, however no changes in the animals injected with L-methionine. Since these changes were measured 5 days following the rapid status change, we propose that our intervention acted systemically and genomically over time to initiate a cascade of changes that ultimately regulate *GnRH1*. We have since shown that this methylation state is characteristic of dominant animals compared with non-dominant animals (Hilliard et al., in preparation) and in future experiments we plan to measure methylation changes at shorter time scales. These data suggest that the methylation modifications related to the immediate act of changing state may act on other sites at shorter time scales.

We also analyzed 3 CPG islands in the upstream region of the GnRH1 gene, where we found no effect on any potential methylation sites from the L-methionine injection (Fig 3C). In total, we examined 34 CPG sites upstream of the GnRH1 gene, including 15 in the promoter and 4 in the coding region. Only those reported here showed methylation differences between manipulated animals five days after treatment.

Our experiment tested the hypothesis that manipulation of methylation state could alter the outcome of fights for social dominance, which it did. We also assessed whether our manipulations had any effect on the GnRH1 gene that is known to respond to social status change in *A*. *burtoni*. Our measurements of epigenetic change at 5 days after status change were limited to GnRH1 related sites where we found two main sites significantly changed. Since animals ascend in status behaviorally almost immediately and modify their reproductive circuit more slowly [3], we propose that methylation changes at shorter time scales may be responsible for the immediate changes in behavior leading to status change. It seems very likely that a much larger difference of gene expression patterns is related to these two phenotypes and discovering where the action is will require genome-wide assessment to understand their collective roles.

## Discussion

We treated animals to modify their DNA methylation chemically and observed the effect on their social status. As described, those animals receiving L-methionine, a methyl donor, had a highly significant likelihood of becoming dominant, while those receiving zebularine, a methylation blocker, had a highly significant likelihood of remaining non dominant. We examined the methylation changes in the GnRH1 gene and found that animals treated with zebularine had higher levels of methylation in a promoter region associated with *egr-1* and in the signal peptide coding regions. We chose the GnRH1 gene for analysis because we know that its expression is directly regulated by social status in that social ascent causes its rapid upregulation [3]. The increased methylation at a GnRH1 promoter and in the coding region is consistent with lower expression of this gene in an animal that does not ascend in rank. The increased methylation induced by zebularine indicates that its action could be regulated by a transgene rather than directly [24]. Interestingly, immediately after the methylation modifying chemicals were injected, we recorded the behavioral response while the measurement of methylation was done at sacrifice five days later, suggesting that methylation states likely changed in the interim.

Our results show that DNA methylation has a major role in establishing social dominance in this vertebrate. Moreover, the treatments appear to change methylation levels of a subset of genes or genomic regions that bias the animal’s chances of becoming dominant. Why might this be the case? To behave in a social system, animals must know their social status and act accordingly. In *A*. *burtoni*, male fish can infer social rank by observation alone [25], a skill shared by human infants who can also mentally represent stable social dominance status [26]. Since environmental changes require complex orchestration of transcription, epigenetic processes such as DNA methylation may provide a mechanism to make a stable short term “memory” of social rank.

In *A*. *burtoni*, changes in rank produce rapid molecular consequences, including up regulation of the immediate early gene and transcription factor *egr-1* within 20 minutes in hypothalamic neurons that regulate downstream targets, including neurons that express gonadotropin-releasing hormone [23]. Social ascent-induced changes can be detected throughout the brain-pituitary-gonadal axis, suggesting that social signals act broadly across the physiological systems of the brain and body [11].

There are several reports of DNA methylation influencing social rank. For example, in comparable experiments to those reported here [27], the activity of DNA-methyltransferase3 (DNMT3), an enzyme that catalyzes the transfer of a methyl group to DNA, was silenced in bee larvae, producing bees with queen-like characteristics. The default worker fate of larvae is regulated via dietary cues. When larvae are fed royal jelly by nursing workers, it globally interferes with the activity of DNMTs leading to demethylation of a core of conserved genes critical for the ontogeny and maintenance of the worker phenotype [28]. Thus DNA methylation as maintained by DNMTs is critical for shaping the worker phenotype and although it affects only a small percentage of the bee’s genes, its activity shifts the animal’s phenotype. This has been further supported using methylome analyses showing that the social division of labor within a colony of bees can be reversibly set through DNA methylation marks within ganglia [8].

Also, in rhesus macaques (*Macaca mulatta*), social rank regulates aspects of the immune system where low status individuals increase gene expression in their immune response to inflammation. The immune response was mediated via the stress response and methylation states of 694 or 70% of the rank related genes were systematically different between high and low-ranking animals [29].

Examining the effects of DNA methylation inhibitors/enhancers in continuous variation in ants where comparable observations resulted in bidirectional control over sizing with similar opposing changes to DNA methylation of the EGFR promoter [30]. Considering that no changes were seen with L-methionine treatment further supports these drugs are acting systemically hypermethylating the genome, suggesting other genes are likely involved.

Injection of DNMT blockers prevents the formation of short-term fear memories [31] and their use after fear conditioning can interfere with the maintenance of long-term cortical memories [32]. L-methionine is a less intrusive chemical than zebularine, since it does not block activity of an enzyme, but rather provides the substrate for methylation and many other biological processes. This is an important distinction since interfering with methylation machinery pharmacologically does not always lead to the same phenotype as one that is generated by naturally lower methylation levels.

Interestingly, fine mapping of methylation within zebularine treated males in the GnRH1 gene showed hypermethylation acting outside of the drug’s known direct mechanism. Zebularine, a nucleoside analog, inhibits the activity of DNA methyltransferases by covalently binding to these enzymes after it is incorporated into DNA [33]. As noted above, differences in DNA methylation were measured 5 days after rapid status change, suggesting our intervention initiated a cascade of changes to regulate *GnRH1* though not in the direction expected. Because neurons in adult animals are not likely to be synthesizing new DNA, it is unclear how zebularine affects methylation levels in these post-mitotic cells but it clearly influences behavior. Since zebularine can inhibit *de novo* methyltransferases independent of replication, this could be the mechanism of action. Zebularine and RG108, a direct DNMT inhibitor [34], have similar behavioral outcomes [35] despite different mechanisms of action. DNMT activity may be required for the maintenance of methylation levels in addition to its role in methylating DNA added during non-mitotic events, such as during repair processes. However, the rapidity with which we see a behavioral effect suggests that DNMT has a more active role during responses to external stimuli.

Our data suggest that DNA methylation could be intimately involved in changes in social status over relatively short time scales although our data do not reveal how this occurs. We hypothesize that this mechanism will include methylation changes in a set of genes related to both the new behavioral phenotype and to the well-described consequent cellular and molecular modifications [36]. For example, ascending in social status produces rapid onset of highly aggressive behavior towards male conspecifics and courtship towards females. Comparing methylomes of dominant and non-dominate males could highlight large changes but also tracking those changes during social ascent could reveal how recognition of social opportunity changes gene expression patterns. It seems likely that epigenetic changes will be found in other contexts where animals have well developed social interactions, including social status. Since social status plays an important role in social behavior across all animals, the role(s) of epigenetic control in its regulation offers opportunities to elucidate how behavioral encounters produce status changes and the genetic bases of its actions.

## Supporting Information

S1 File (Fig A). Aquaria for raising Never Been Dominant (NBD) males. 10 NBD males were raised from 2 weeks post-fertilization in community tanks with 4 large suppressor D males and 4 females. 2 spawning shelters were placed in the tank to limit the number of mating territories available. (Fig B). Experimental aquaria. Two size-matched males were injected and placed in middle experimental compartment with 3 smaller females. On either side were community compartments separated by clear barriers with 2 smaller males and 3 smaller females allowing behavioral interactions through transparent barriers. (Fig C). Data comparing control and L-Methionine injected animals. Averaged sum of daily territorial behaviors with non-territorial behaviors subtracted, comparing L-methionine (blue) and vehicle-injected animals (dashed). N = 8 for each group. (Fig D). Data comparing control and zebularine injected animals. Averaged sum of daily territorial with non-territorial behaviors subtracted, comparing zebularine (red) and vehicle-injected animals (dashed). N = 8 for each group. (Fig E). Brain staining for methylation. Coronal brain sections from *A*. *burtoni* male brains in the region of the pre-optic area stained with a monoclonal antibody specific for methylated cytosine residues for the four treatment groups (L-methionine and its controls; Zebularine and its controls. Top row (5mC); Middle row (Dapi counterstain); Bottom row (merged images).(Fig F). Comparison of fluorescence intensities for Fig E. Fluorescence intensity in 5-mC stained cells (N = 40) in POA brain sections from zebularine, methionine, and control injected animals. There was no statistically significant difference in flurorescence intensity between any of the treatment groups.
(DOCX)
