# Supplementary material for: Epigenetic DNA Methylation Linked to Social Dominance
Source: PLoS One. 2015 Dec 30;10(12):e0144750. doi: 10.1371/journal.pone.0144750 (PMC4696829; doi:10.1371/journal.pone.0144750)
Supplement: S1 File — (Fig A). Aquaria for raising Never Been Dominant (NBD) males. 10 NBD males were raised from 2 weeks post-fertilization in community tanks with 4 large suppressor D males and 4 females. 2 spawning shelters were placed in the tank to limit the number of mating territories available. (Fig B). Experimental aquaria. Two size-matched males were injected and placed in middle experimental compartment with 3 smaller females. On either side were community compartments separated by clear barriers with 2 smaller males and 3 smaller females allowing behavioral interactions through transparent barriers. (Fig C). Data comparing control and L-Methionine injected animals. Averaged sum of daily territorial behaviors with non-territorial behaviors subtracted, comparing L-methionine (blue) and vehicle-injected animals (dashed). N = 8 for each group. (Fig D). Data comparing control and zebularine injected animals. Averaged sum of daily territorial with non-territorial behaviors subtracted, comparing zebularine (red) and vehicle-injected animals (dashed). N = 8 for each group. (Fig E). Brain staining for methylation. Coronal brain sections from A. burtoni male brains in the region of the pre-optic area stained with a monoclonal antibody specific for methylated cytosine residues for the four treatment groups (L-methionine and its controls; Zebularine and its controls. Top row (5mC); Middle row (Dapi counterstain); Bottom row (merged images).(Fig F). Comparison of fluorescence intensities for Fig E. Fluorescence intensity in 5-mC stained cells (N = 40) in POA brain sections from zebularine, methionine, and control injected animals. There was no statistically significant difference in flurorescence intensity between any of the treatment groups. (DOCX) [file pone.0144750.s001.docx]

**Supporting Information**

**Animals**

Subjects were laboratory-bred cichlid fish, *Astatotilapia burtoni*, derived from wild-caught stock collected in Lake Tanganyika, Africa (1). Animals were housed in aquaria under conditions that closely mimicked their natural habitat (28°C, pH 8.0, 12 hours light and 12 hours dark cycle with full spectrum illumination, and constant aeration), and fed daily with cichlid pellets and flakes (AquaDine, Healdsburg, CA). Animals were raised from hatching and initially fed ground flakes smaller than the width of their mouths. From one month to three months post fertilization, their diet was supplemented with brine shrimp to increase their growth rate. Experimental animals were fully developed adults three months post-fertilization and were fed approximately one pellet and one flake per adult fish thereafter. Aquaria contained gravel substrate and hemi-sected terra-cotta flowerpots that served as spawning shelters. All animal experiments were approved by the Stanford University’s Institutional Animal Care and Use Committee (IACUC protocol 9882.

**Social Manipulations**

Dominant vs. non-dominant males

In their natural habitat, adult male *A. burtoni* live as one of two distinct behavioral phenotypes that reflect their position in the social hierarchy: Dominant (D) males have bright coloration, are reproductively capable, and defend spawning territories. Non-dominant (ND) males have a dull grey coloration that blends with the substrate, school with females and are not reproductively capable (1,2). Males establish their social status by engaging in aggressive, territorial behaviors towards other males and reproductive behaviors towards females (1). In the laboratory, the ratio of territorial to non-territorial males can be controlled by adjusting the size of the aquaria relative to the size of the fish and the availability of spawning shelters (2).

**Raising animals that have never been dominant (NBD)**

Ten clutches of juvenile animals, aged ~4 weeks prior to expression of any of the well described hallmarks of social dominance (2), were reared separately in 151 liter tanks containing 4 large dominant males and two spawning shelters for a minimum of 2 months (3) (See S Fig A). Each tank was observed three times per week for 5 minutes at a time for a minimum of 4 weeks and any juvenile males displaying any feature characteristic of the dominant phenotype (2) were removed from the experiment. Only males between 5 and 6 cm in length with clear NBD behavioral phenotypes were used in the experiment.


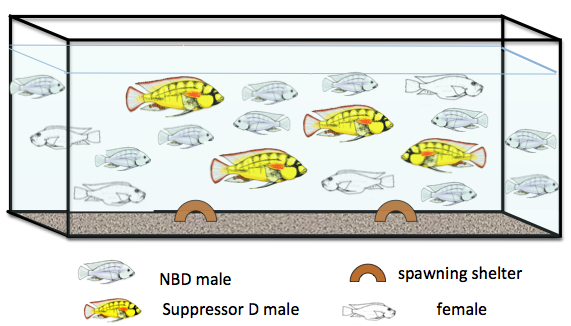


**Fig A**: **Aquaria for raising Never Been Dominant (NBD) males.** 10 NBD males were raised from 2 weeks post-fertilization in community tanks with 4 large suppressor D males and 4 females. 2 spawning shelters were placed in the tank to limit the number of mating territories available.

**Behavioral Paradigm**

When two NBD males of equal size are placed together in an aquarium large enough to sustain only one territory, fighting begins almost immediately and in a short time (<< 30 minutes), one male becomes dominant over the other. The dominant male engages in aggressive behaviors toward the non-dominant male, and directs aggressive behaviors toward males in adjoining enclosures separated by clear plastic barriers (S Fig B), and mating behaviors toward females. The non-dominant male typically flees or remains in the upper part of the water column. All these behaviors were characteristic of animals injected with either experimental reagent (L-methionine or zebularine) or those injected with the control substance (vehicle). Initially, behaviors were recorded for 20 minutes at three different times during the day – 10am, 2pm, and 6pm (TRENDnet internet camera, Torrance, CA). Behaviors of the animals at all three time points were not significantly different so behavioral data were collected at 2 PM. Behaviors were recorded daily for 20 minutes at 2pm for 5 days post-injection after which the experimental animals were sacrificed and their brains were removed and immediately frozen in O.C.T. (Microm HM550, Thermo Scientific, Waltham, MA). Only experimental pairs in which one male attained clearly dominant social status and the other retained non-dominant social status, as determined by behavioral and physiological measurements were used in the final data analysis.


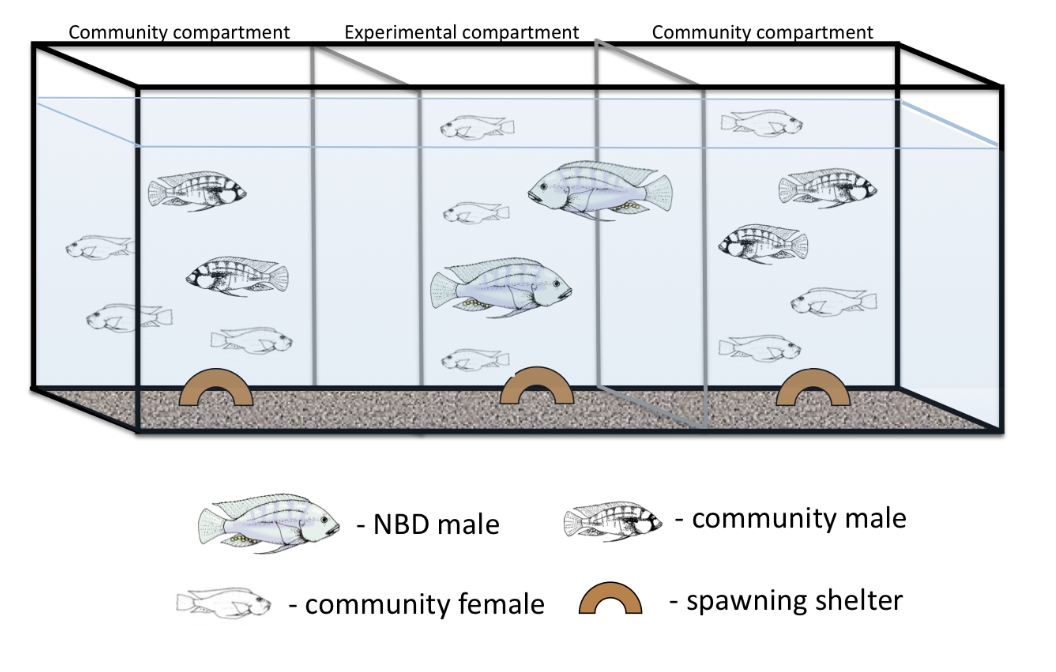


**Fig B: Experimental aquaria**. Two size-matched males were injected and placed in middle experimental compartment with 3 smaller females. On either side were community compartments separated by clear barriers with 2 smaller males and 3 smaller females allowing behavioral interactions through transparent barriers.

**Methionine and Zebularine Injections**

As described in the Experimental Design section of the main text, the goal of these experiments was to test whether modifying the methylation state of animals could influence the outcome of fights over social dominance. To do this, pairs of NBD males (2N=33) were selected from rearing tanks. Pairs were carefully matched for size to within 0.2 cm in length and within 0.5g in weight to ensure that relative size was not a factor in determining the social hierarchy. Individuals in each pair were taken from separate tanks to ensure that the experimental animals had not experienced social interactions with each other previously (4). Each animal received an intraperitoneal injection of either the vehicle (30% DMSO dissolved in distilled water) or one of two drugs: L-methionine or zebularine. For L-methionine, a methylating agent, L-methionine (Sigma; 25mg/ml 30% DMSO) was intraperitoneally injected at a dose of 200µg/g. For zebularine (Sigma or Tocris Chemicals), 200µg/g body weight, a DNA methytransferase inhibitor dissolved in a 30% DMSO/ ddH_2_O solution. Experimental procedures were carried out double-blind so that neither the individuals injecting nor observing knew which animals received which treatment. Clipping a small bit from the dorsal fins in a distinctive pattern marked individuals, allowing unambiguous subsequent animal identification.

Immediately after injection, males were placed together in a 120 L tank for the following five days. To simulate the semi-natural environment in which they usually live, community fish were placed across a clear barrier on both sides of the aquarium containing the males being tested (5). These community fish, both males and females, were smaller than the two test subjects so that no large, aggressive male was present to threaten the test subjects. There were also three small females in the test-subject compartment to provide an incentive for competition between the males to become dominant and to add courting behaviors to the general repertoire of aggressive behaviors for the behavioral analysis.

**Control Injections**

Control experiments were performed exactly as above, except that both animals were injected with the vehicle control (30% DMSO / ddH2O solution). Control-injected D animals were compared to L-methionine-injected D animals to ensure that subjects in both groups had similar behaviors (S Fig C). There was no significant difference between methionine-injected and control-injected D animals F(1,63) = 1.32, p = 0.26 There was no significant difference in behavior between individual days (F(3,63) = 0.20, p = 0.89) nor significant interaction between injection treatment and individual days (F(3,63) = 0.9077, p = 0.44). Correspondingly, control-injected ND animals were compared to zebularine-injected ND animals to ensure that subjects in both groups had similar behaviors (S Fig D). There was no significant difference between zebularine-injected and control-injected ND animals F(1,79) = 1.26, p = 0.27. There was no significant difference in behavior between individual days (F(4,79) = 1.77, p = 0.14) or significant interaction between injection treatment and individual days (F(4,79) = 0.24, p = 0.92).


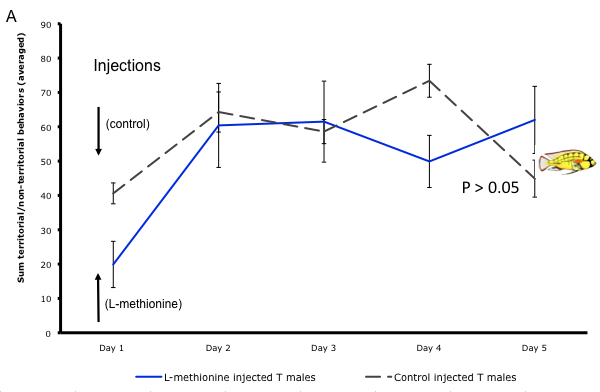


**Fig C:** Averaged sum of daily territorial behaviors with non-territorial behaviors subtracted, comparing L-methionine (blue) and vehicle-injected animals (dashed). N = 8 for each group.


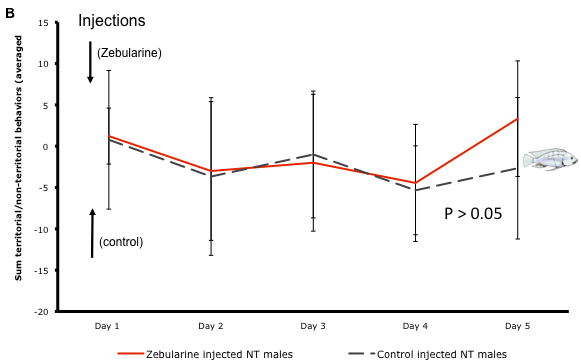


**Fig D:** Averaged sum of daily territorial with non-territorial behaviors subtracted, comparing zebularine (red) and vehicle-injected animals (dashed). N = 8 for each group.

**Behavioral Observations**

After subject animals were placed together in the experimental tank, their behaviors were recorded remotely via webcams mounted directly in front of each experimental tank. Through pilot experiments described above, we established that a 5-minute daily interval provided a sufficiently representative sample of the test animals’ behaviors. Behaviors were recorded daily for a 20-minute interval of which the first 5 minutes were scored separately for each male. Behaviors were scored using a previously established scoring paradigm quantifying characteristically dominant behavior and non-dominant behaviors in males (6). The following behaviors dominant behaviors were coded: Approach, Chase, Frontal Threat, Lateral Display, Border Fight, Court/Quiver, Lead/Waggle, Dig, Spawn site entry, Bite, Chafe. Fleeing was counted as a non-dominant behavior. To obtain a single score for each behavioral period, each dominant behavior performed was assigned 1 point and added to the overall score, and for each time a non-dominant behavior was performed (i.e. fleeing) 1 point was subtracted from the overall score. The only exception was spawn site entry, for which 2 points were added to the score due to its fairly rare occurrence and it being a strong indication the an animal has complete dominance over the territory. Dominant animals achieved a score of at least 25 per 5-minute interval.

**Animal Sacrifice**

Five days after the injection of L-methionine, zebularline or control injections, the animals were sacrificed by rapid cervical transection and the relative gonad size measured (gonadosomatic index; GSI = [gonad mass/body mass] x 100). Based on prior work, ND animals have a GSI in the range of 0.1 to 0.5 and D animals have a GSI range of 0.5 to 1.0 (3), however, for these animals we used behavior, coloration as well as GSI to classify the animals so that ND animals had a GSI between 0.2 and 0.6, while D animals have a GSI between 0.4 and 0.8.

**Tissue Preparation**

Standard length (± 1mm) and weight (± 0.01 g) were measured and animals sacrificed by cervical transection within 2 minutes of capture. Whole brains were removed intact from the braincase via dissection, mounted in molds filled with optimal cutting temperature mounting medium (Neg50; Thermo Scientific, Waltham, MA), flash frozen in a methanol/dry ice bath, and stored at -80º C until histological sectioning. Gonads were removed and weighed (± 0.01g) to calculate GSI.

**Immunocytochemistry**

To quantify levels of global methylation in the pre-optic area (POA) of the hypothalamus, the methylation sites were localized with antibody staining. Frozen brains from zebularine injected (ND), methionine injected (D) and control injected animals (ND and D) were sectioned coronally into 30µm sections using a cryostat (Microm HM550, Thermo Scientific, Waltham, MA), placed on cooled glass microscope slides and stored at -80º C. Sections from each of the four brain types to be analyzed were placed in alternating order in each slide, so that at least 6 sections from each brain type were on each slide. Sections were fixed in cold methanol for 3 minutes at room temperature and rinsed with dH2O for 1 minute. Tissue was denatured in 2N HCl for 2 hours at room temperature and then rinsed again in dH2O for 1 minute. To block non-specific staining, cells were incubated for 2 hours at room temperature in a blocking buffer made of 0.2% BSA, 1X PBS, and 10% normal goat serum. Then primary antibody specific for methylated cytosine residues made from monoclonal mouse antibody (Abcam, Cambridge, MA) was added to sections at a 1/300 dilution, which then was incubated for 36 hours at 4ºC. A secondary antibody (Alexa-Fluor 488, Invitrogen, Carlsbad, CA) and DAPI nuclear stain (Sigma, St.Louis, MO) were added at a 1/300 and 1/5000 dilution, respectively, and incubated for 2 hours at room temperature before a final rinse in PBS. Slides were mounted using Fluoromount aqueous mounting medium (Diagnostic Biosystems, Pleasanton, CA). Multiple images of each section were taken using a SPOT camera (Diagnostic Instruments, Inc., Sterling Heights, MI) on a Zeiss microscope (Oberkochen, Germany) at a magnification of 67X. (S Fig E).

Fluorescence intensity was calculated by measuring corrected total cell fluorescence as described in (7). To identify the particles to be analyzed, images were viewed in the channel that showed methylation staining and were thresholded to greyscale values between 600 and 4095 (with 0 being pure white and 4095 being pure black) (Image J, NIH) . These particles were compared to the channel with DAPI staining only particles that showed fluorescence in both channels were analyzed for relative methylation levels. An outline was drawn around 40 selected cells for each treatment, which were measured for area, integrated density and mean grey value. Three regions of no fluorescence were measured adjacent to each section to obtain mean background fluorescence. Corrected total cell fluorescence (CTCF) was calculated by the following formula: CTCF = Integrated Density – (Area of selected cell X mean background fluorescence). There was no statistically significant difference in staining intensity between L- methionine and control injected D animals (*t(*78) = 1.99, p = 0.30), zebularine and control injected ND animals (*t(*77) = 1.99, p = 0.25), L-methionine and zebularine injected animals (*t(*69) = 1.99, p = 0.19), or control injected D and ND animals (*t(*72) = 1.99, p = 0.40), This indicating that global methylation levels were unaffected by social status or L-methinonine or zebularine treatment. (S Fig F)


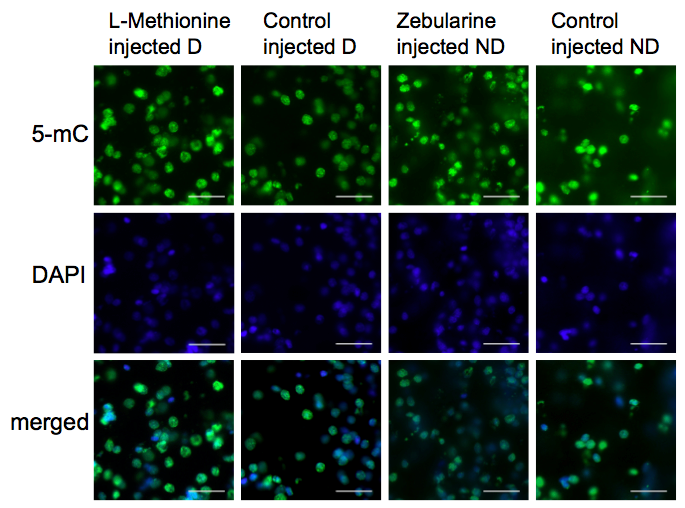


**Fig E:** **Brain staining for methylation.** Coronal brain sections from *A. burtoni* male brains in the region of the pre-optic area stained with a monoclonal antibody specific for methylated cytosine residues for the four treatment groups (L-methionine and its controls; Zebularine and its controls. Top row (5mC); Middle row (Dapi counterstain); Bottom row (merged images).

**Fig F: Comparison of fluorescence intensities for Fig E.** Fluorescence intensity in 5-mC stained cells (N=40) in POA brain sections from zebularine, methionine, and control injected animals. There was no statistically significant difference in flurorescence intensity between any of the treatment groups.

**Statistical analyses**

Behavioral scores computed as described above, for each day and for each animal were compared using two-factor analysis of variance (ANOVA) with replication. Differences in social outcomes for zebularine and L-methionine injected fish were analyzed with a chi-square test (Fig 1). Relative fluorescent intensity was compared using 2-tailed unpaired Student’s t-test.

For analysis of methylation at individual CpG sites, data are presented as percent methylation averaged across samples. Differences in DNA methylation between groups were statistically analyzed as described in Bock (8) by performing unpaired, one-tailed t-tests in both directions and using the generated p values to calculate q values as estimates of the multiple-testing-correction false discovery rate (FDR; 9, 10). Due to the sample sizes in this study, the bootstrap method was used (11) and λ was set to the single value of 0 (12, 13).

To compare methylation across genomic regions, CpG methylation values were combined and averaged across samples. Differences in DNA methylation between groups were calculated by performing unpaired, one-tailed t tests in both directions and combining the generated p values using Fisher’s method (14). P values obtained for separate genomic regions were used to calculate q values as described above. Combining CpG methylation data in this matter increases statistical power as neighboring CpGs exhibit correlated changes.

T tests were performed using R (ver. 2.15.1). Q values were also calculated using the R (QVALUE package, ver. 1.0). Combined Fisher p values were calculated using the MetaP (<http://compute1.lsrc.duke.edu/softwares/MetaP/metap.php>).

**References:**

1) Fernald, RD, Hirata, NR. Field-Study of *Haplochromis burtoni* - Quantitative Behavioral Observations. Anim Behav. 1977; 25(4):964-975.

2) Fraley, NB, Fernald, RD. Social-Control of Developmental Rate in the African Cichlid, *Haplochromis burtoni*. Zeitschrift für Tierpsychologie-Journal of Comparative Ethology. 1982; 60(1): 66-82.

3) Davis MR, Fernald RD. Social control of neuronal soma size. Journal of neurobiology. 1990;21(8):1180-8.

4) Grosenick L, Clement TS, Fernald RD. Fish can infer social rank by observation alone. Nature. 2007;445(7126):429-32.

5) Burmeister SS, Jarvis ED, Fernald RD. Rapid behavioral and genomic responses to social opportunity. PLoS biology. 2005;3(11):e363.

6) Fernald, RD. Quantitative behavioural observations of *Haplochromis burtoni* under semi‑natural conditions. Anim Beh.1977; 25:643-653.

7) McCloy, R. A., Rogers, S., Caldon, C. E., Lorca, T., Castro, A., and Burgess, A. Partial inhibition of Cdk1 in G 2 phase overrides the SAC and decouples mitotic events. Cell Cycle, 2014. 13: 1400–1412

8) Bock, C., Analysing and interpreting DNA methylation data. Nat Rev Genet, 2012. 13(10): p. 705-19.

9) Storey, J.D. and R. Tibshirani, Statistical methods for identifying differentially expressed genes in DNA microarrays. Methods Mol Biol, 2003. 224: p. 149-57.

10) Storey, J.D. and R. Tibshirani, Statistical significance for genomewide studies. Proc Natl Acad Sci U S A, 2003. 100(16): p. 9440-5.

11) Storey, J.D., J.E. Taylor and D. Siegmund Strong control, conservative point estimation and simultaneous conservative consistency of false discovery rates: a unified approach *J. R. Statist. Soc.* B (2004) **66**, *Part* 1, *pp.* 187–205

12) Benjamini, Y. and Y. Hochberg, Controlling the False Discovery Rate: A Practical and Powerful Approach to Multiple Testing. Journal of the Royal Statistical Society. Series B (Methodological), 1995. 57(1): p. 289-300..

13)  Storey, J.D., A direct approach to false discovery rates. Journal of the Royal Statistical Society: Series B (Statistical Methodology), 2002. 64(3): p. 479-498.

14) Fisher, R. A., 1932. [*Statistical Methods for Research Workers*](http://en.wikipedia.org/wiki/Statistical_Methods_for_Research_Workers), 4th Edition. Oliver and Boyd, Edinburgh.
